# Human post-mortem synapse proteome integrity screening for proteomic studies of postsynaptic complexes

**DOI:** 10.1186/s13041-014-0088-4

**Published:** 2014-11-28

**Authors:** Àlex Bayés, Mark O Collins, Clare M Galtrey, Clémence Simonnet, Marcia Roy, Mike DR Croning, Gemma Gou, Louie N van de Lagemaat, David Milward, Ian R Whittle, Colin Smith, Jyoti S Choudhary, Seth GN Grant

**Affiliations:** Molecular Physiology of the Synapse Laboratory, Biomedical Research Institute Sant Pau (IIB Sant Pau), Sant Antoni Mª Claret 167, 08025 Barcelona, Spain; Universitat Autònoma de Barcelona, 08193 Bellaterra, Cerdanyola del Vallès, Spain; Department of Biomedical Science, The Centre for Membrane Interactions and Dynamics, University of Sheffield, Western Bank, Sheffield, S10 2TN UK; Department of Neurology, St George’s Hospital, London, UK; Genes to Cognition Programme, Molecular Neuroscience, Centre for Clinical Brain Science and Centre for Neuroregeneration, University of Edinburgh, Edinburgh, EH16 4SB UK; Linguamatics, 324 Cambridge Science Park, Cambridge, CB4 0WG UK; Academic Department of Neuropathology, Centre for Clinical Brain Sciences, University of Edinburgh, Edinburgh, UK; Proteomic Mass Spectrometry, The Wellcome Trust Sanger Institute, Hinxton, Cambridgeshire UK

**Keywords:** Synapse, Proteomics, Mass spectrometry, Supercomplex, Post-mortem brain, MAGUK, Psychiatric disorder

## Abstract

**Background:**

Synapses are fundamental components of brain circuits and are disrupted in over 100 neurological and psychiatric diseases. The synapse proteome is physically organized into multiprotein complexes and polygenic mutations converge on postsynaptic complexes in schizophrenia, autism and intellectual disability. Directly characterising human synapses and their multiprotein complexes from post-mortem tissue is essential to understanding disease mechanisms. However, multiprotein complexes have not been directly isolated from human synapses and the feasibility of their isolation from post-mortem tissue is unknown.

**Results:**

Here we establish a screening assay and criteria to identify post-mortem brain samples containing well-preserved synapse proteomes, revealing that neocortex samples are best preserved. We also develop a rapid method for the isolation of synapse proteomes from human brain, allowing large numbers of post-mortem samples to be processed in a short time frame. We perform the first purification and proteomic mass spectrometry analysis of MAGUK Associated Signalling Complexes (MASC) from neurosurgical and post-mortem tissue and find genetic evidence for their involvement in over seventy human brain diseases.

**Conclusions:**

We have demonstrated that synaptic proteome integrity can be rapidly assessed from human post-mortem brain samples prior to its analysis with sophisticated proteomic methods. We have also shown that proteomics of synapse multiprotein complexes from well preserved post-mortem tissue is possible, obtaining structures highly similar to those isolated from biopsy tissue. Finally we have shown that MASC from human synapses are involved with over seventy brain disorders. These findings should have wide application in understanding the synaptic basis of psychiatric and other mental disorders.

**Electronic supplementary material:**

The online version of this article (doi:10.1186/s13041-014-0088-4) contains supplementary material, which is available to authorized users.

## Background

Synapse proteomics involves the comprehensive analysis of individual synapse proteins and their organization and assembly into multiprotein complexes [1]. Proteomic mass spectrometry analysis of mammalian synapses demonstrated that synapse proteomes are highly complex with over 1000 proteins in humans [2,3]. This knowledge, combined with human genetic studies, enabled the systematic identification and classification of human diseases involving synapse proteins, which are now referred to as Synaptopathies [4]. Several reports [5-9] show it is feasible to profile individual proteins in the human postsynaptic density (PSD) proteome from post-mortem tissue and three studies in Alzheimer’s Disease [7,8] and alcohol addiction [9] show the potential for direct molecular studies of synaptopathy.

To date there are no proteomic studies of synaptic multiprotein complexes from either post-mortem (PM) or neurosurgical biopsy (NSB) material. In mice virtually all synapse proteins are assembled into supramolecular complexes, some of which are in turn assembled into supercomplexes [10,11]. These can be isolated using a variety of affinity purification methods including peptides, chemical ligands, antibodies and genetically encoded affinity tags [4,12,13]. The prototype postsynaptic supercomplexes are known as MASC (MAGUK Associated Signalling Complexes) and they are organized around scaffolding proteins encoded by the DLG/MAGUK gene family. Humans and mice have 4 DLG paralogues: DLG1/SAP97, DLG2/PSD93, DLG3/SAP102 and DLG4/PSD95. MASCs were originally characterized from mouse brain and are ~2MDa in size, containing, among other proteins, glutamate receptors, signalling proteins and potassium channels [12,14]. The importance of MASCs for cognition is emphasized by human genetic studies of mental disorders including schizophrenia, autism, intellectual disability and other diseases, which show mutations in MASC components [15-18]; similarly, mutations of MASC genes in mice also result in cognitive impairments [4,19-21].

To better understand synaptic biochemical variations in health and disease, the ability to isolate and characterize MASC from PM human brain is critical. Unfortunately, the greatest obstacle to human synapse proteomics is the protein degradation that occurs due to ante- and post-mortem conditions. Although brain banks report indices for assessing sample quality such as PM time interval, tissue pH and an RNA integrity index (RIN), their relevance for proteome integrity is unknown. Here we have surveyed the variability and suitability of PM tissue with the purpose of isolating and characterizing postsynaptic protein complexes using proteomics. We report that standard brain quality indicators from tissue banks poorly predict the integrity of the postsynaptic proteome and we have developed a new index that is a robust correlate of synapse proteome integrity allowing proteomic analysis of MASC from PM samples. These studies and their data resources reveal the importance of human MASC in a large number of human diseases. The new approaches will also facilitate isolation of other synapse multiprotein complexes and their direct study in diseased tissue. All the data generated in this study is freely available in the G2Cdb database (http://www.genes2cognition.org/publications/human-masc).

## Results and discussion

### Rapid isolation of postsynaptic fractions from small PM samples

**Figure 1:**
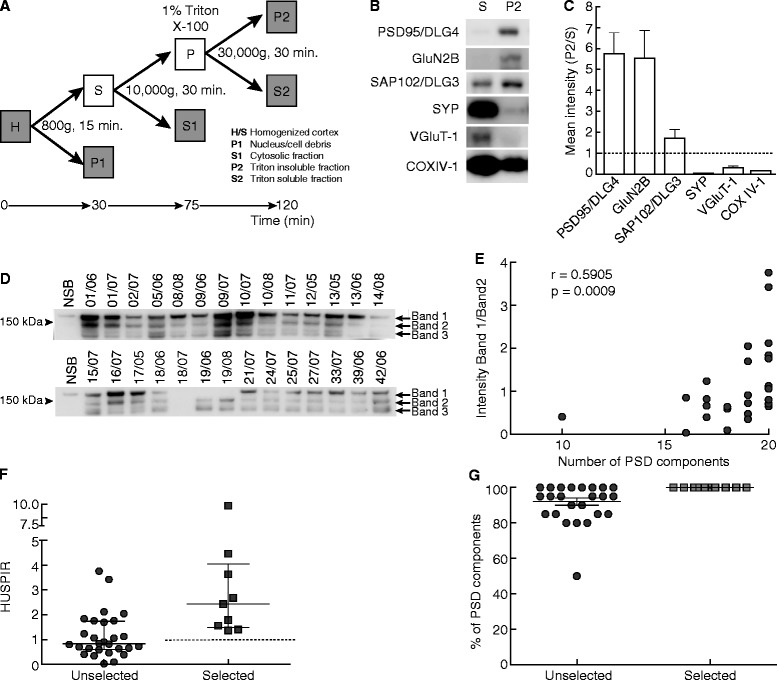
**Method for rapid isolation of synaptic-enriched fractions from PM human cortex. GluN2B degradation and correlation with number of intact PSD proteins. A**. Schematic representation of the subcellular fractionation method used to obtain postsynaptic protein enriched fractions (P2). Procedure time is indicated. H and S, homogenized cortex; P1, nucleus/cell debris; S1, cytosolic fraction; P2, triton insoluble fraction; S2, triton soluble fraction. **B**. Immunoblot showing protein enrichment or depletion between sample S and P2 from isolation protocol described in A. Postsynaptic markers: PSD95/DLG4, GluN2B and SAP102/DLG3. Pre-synaptic-markers: SYP and VGluT-1. A marker of mitochondria is also included: COXIV-1. **C.** Mean fold enrichment of proteins in final P2 fraction compared to starting S fraction analysed by immunoblotting (n = 5). Postsynaptic proteins were enriched in P2, while presynaptic and mitochondrial markers were depleted (S/P2 < 1). **D**. GluN2B immunoblot from control NSB and 28 PM samples (Additional file 1). PM samples show three main bands, band 1 corresponding with the full-length protein. The ratio of band 1 over band 2 intensities provides HUSPIR ratio. The antibody used was designed against the C-terminal region of GluN2B (BD Bioscience ref. 610416). **E**. For each PM sample the HUSPIR ratio is plotted against the number of intact PSD proteins. Significant positive Spearman’s coefficient of correlation (r) and p-value (p) are indicated. **F**. HUSPIR ratio for the set of 28 unselected samples and the set of 9 samples selected. Median and interquartile range shown. **G**. Comparison between percentage of PSD components observed in the set of 28 unselected samples and the prospective set of 9 selected samples. Median and interquartile range shown.

Our initial objectives were to: i) enhance sample preparation speed to improve preservation of protein complexes and allow greater numbers of samples to be analysed, and ii) increase protein yield enabling studies on small amounts of tissue. We reduced the duration of the isolation of postsynapse-enriched fractions from 6–8 h [22] to 2 hours by removing the density gradient fractionation step and maintaining the well-known non-ionic detergent insolubility of the PSD of excitatory synapses (Figure 1A, see methods). The high protein yield (2.8 ± 0.13 μg SEM, n = 35 protein in P2 fraction/mg of tissue) of this protocol enabled a 10-fold reduction in the amount of starting material permitting routine isolation of synaptic-enriched structures from as little as 100 mg of human brain tissue. Enrichment of synapse proteins in the P2 fraction was confirmed (Figure 1B,C).

Next, we applied this protocol to 28 randomly selected PM brains from the MRC Edinburgh Brain Bank (Additional file 1) and assessed the degradation of 20 important postsynaptic proteins using immunoblotting (Additional file 2: Figure S1). Comparison with control NSB tissue (Additional file 2: Figure S1) permitted counting and scoring of the integrity of each protein in each PM sample. The correlation coefficient for the number of intact proteins in each sample compared with Brain Bank quality indicators including RNA integrity, time since death and other measures (Table 1) was calculated. We failed to find statistical significance in any of these correlations, indicating these measures do not reliably predict synapse proteome integrity; which triggered us to develop a new method to evaluate synaptic proteome integrity that could be readily applied to PM samples.

**Table 1:** **Correlation between brain bank tissue quality parameters and number of detected postsynaptic density proteins**

**Parameter**	**Spearman r**	**p value (two-tailed)**
PM Interval *(n = 28)*	0.01079	0.9566 (n.s.)
pH *(n = 25)*	−0.075	0.1193 (n.s.)
RNA Integrity Number (RIN) *(n = 20)*	0.1193	0.6163 (n.s.)
Intensity of GluN2B Band 1/Band 2 *(n = 28)*	0.5905	0.0009 (***)
Intensity of GluN2B Band 1/Band 3 *(n = 28)*	−0.01091	0.956 (n.s.)
Intensity of GluN2B Band 1/Band 2 + Band 3 *(n = 28)*	0.4831	0.0092 (**)

Number in brackets indicates the number of tissue samples (out of 28) with a value for that particular parameter; n.s. not significant; ***p < 0.001 and **p < 0.01.

### A simple screening method to measure PM synapse proteome integrity

Inspection of these 20 immunoblots (Additional file 2: Figure S1) revealed that PM samples in GluN2B (2B subunit of the N-methyl-D-aspartate glutamate receptor) presented two low-molecular weight bands (see bands 2 and 3 on Figure 1D) that corresponded with GluN2B proteolysis products due to PM conditions as described previously [23]. These degradation products, which are absent from biopsy samples (NSB, Figure 1D), are already apparent 2 h after death [23]. Different PM samples showed different intensities for these three bands; for instance, some samples lacked all three bands (sample 18/07) indicating complete degradation of the receptor and some lacked only the uppermost Band 1 (samples 19/06 or 19/08) indicating partial degradation. To estimate the extent of degradation of each sample, the ratio of Band 1/Band 2 intensity was measured for all 28 samples (median ratio: 0.83). We next correlated these values with the counts of present immunoblotted PSD proteins (Additional file 2: Figure S1) and in contrast to what was observed with brain bank indicators, we found significant positive correlation (Spearman’s rho = 0.59, P <0.001, Figure 1E). These results suggest this ratio can be used to estimate synaptic proteome preservation in PM brain samples and hereafter we refer to this ratio as HUSPIR (HUman Synapse Proteome Integrity Ratio).

To validate the usefulness of HUSPIR we prospectively screened another group of nine PM samples (Selected PM Group) that were presumed to be of high quality since they were obtained from individuals who died suddenly, before the age of 60, had a short agonal state and PM interval, and showed no detectable neuropathological abnormalities. The median HUSPIR value for this group (2.43, Figure 1F) was significantly higher (p = 0.001; Mann Whitney Test) than the median HUSPIR for the initial set (Unselected PM Group, Figure 1F). As predicted by HUSPIR the selected group presented all PSD components analysed by immunoblotting (Figure 1G and Additional file 2: Figure S2). These results confirm that the simple and rapid measurement of HUSPIR, based on GluN2B band ratios, reliably predicts synapse proteome integrity. Based on these results, we propose using PM samples with a HUSPIR >1 for biochemical evaluation.

### Synaptic proteome integrity varies between cortical and other brain regions

**Figure 2:**
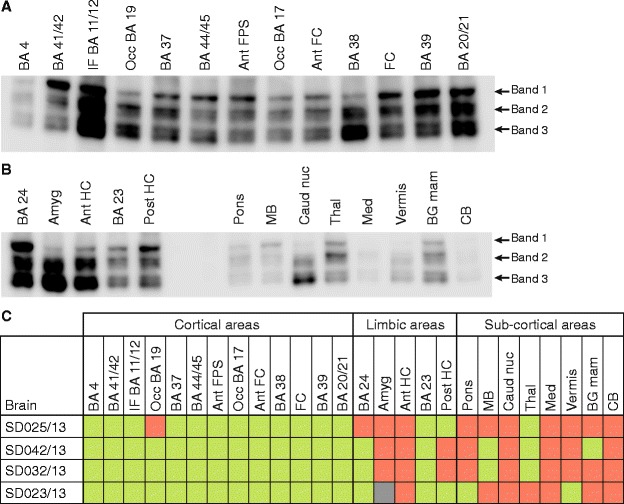
**Comparative GluN2B degradation between brain areas. A**. Representative immunoblot of GluN2B for 13 different cortical regions from brain SD025/13 (Additional file 1). These cortical regions included the Broadmann Areas (BA) 4, 11/12, 17, 19, 20/21, 37, 38, 39, 41/42 and 44/45 (Broca’s area), as well as three frontal cortex samples: frontal convexity (FC), anterior frontal convexity (Ant FC) and anterior frontal parasagital (Ant FPS). GluN2B degradation bands are indicated. **B**. Representative immunoblot of GluN2B for 5 limbic areas: Broadmann areas (BA) 23 and 24, anterior and posterior Hipocampus (HC) and amygdala (Amyg); and 8 sub-cortical regions: Pons, Midbrain (MB), Caudate nucleus (Caud nuc), Thalamus (Thal), Medulla (Med), Vermis, Basal ganglia mammillary body (BG mam) and Cerebellum (CB). GluN2B degradation bands are indicated. **C**. Plot summarizing HUSPIR values for 26 brain areas (13 cortical, 5 limbic and 8 sub-cortical) analysed by immunoblot for GluN2B degradation in 4 different brains (SD025/13, SD042/13, SD032/13 and SD023/13). HUSPIR values above one are indicated in green and below in red. Grey indicates no measure could be taken.

We were also interested in evaluating if postsynaptic proteome integrity was homogenous between different areas of the same brain. To address this issue we isolated postsynaptic densities (PSD) from 26 different brain regions in 4 different post-mortem brains from the ‘sudden-death brain bank collection’ (Edinburgh MRC-Brain Bank). These regions included 13 cortical areas, 5 limbic structures and 8 sub-cortical regions (Figure 2). Each isolated PSD fraction was subjected to GluN2B immunoblot (Figure 2A,B) to determine its corresponding HUSPIR value.

As summarized in Figure 2C HUSPIR values varied between cortical and other brain regions. While most HUSPIR values for cortical regions were above 1, suggesting a good preservation of its postsynaptic proteome, other brain regions showed a HUSPIR <1 in more than half of the samples analysed (34/52; Figure 2C). The fact that limbic areas anatomically close to the cortex (cingulate gyrus: BA23 and BA24) show high HUSPIR values in the 4 brains analysed gives further evidence for a better preservation of the synaptic proteome in more anterior brain regions. Finally, this data also indicates that when working with non-cortical brain areas HUSPIR should be measured for each brain sample included in the study. The HUSPIR value of one cortical region has a good predictive value for other cortical regions of the same brain, as shown in Figure 2C.

### Isolation and proteomics of human MAGUK associated signalling complexes

**Figure 3:**
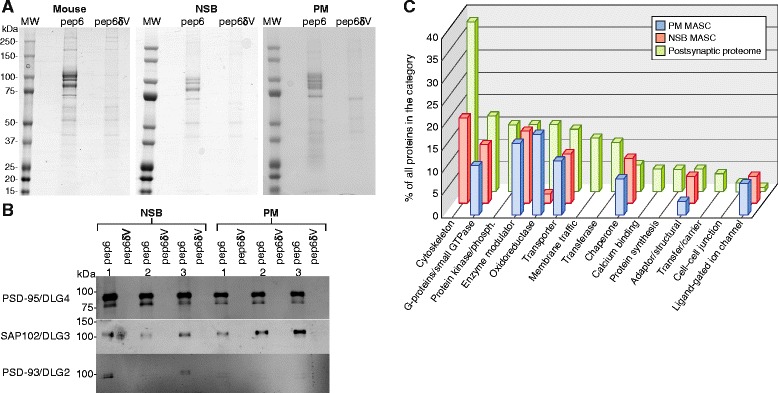
**Affinity purification of the MAGUK Associated Signalling Complexes (MASC) from brain cortex and observed MASC composition in NSB or PM frontal cortex. A**. Coomassie stained SDS-PAGE electrophoresis of MASC complexes isolated by affinity purification from mouse cortex, human NSB and PM frontal cortex. Human sample details in Additional file 1. MW: Molecular Weight markers; pep6, eluate from affinity purification using pep6 peptide; pep6δV, eluate from affinity purification using control peptide. **B**. Immunoblots of three members of the DLG4/PSD-95 family (DLG4/PSD-95, DLG3/SAP102 and DLG2/PSD-93) for 6 human MASC purifications (pep6), and corresponding controls (pep6δV); three from NSB and three from PM frontal cortex. **C**. Panther Protein Classes in the postsynaptic proteome (in green), the NSB MASC (in red) or the PM MASC (in blue) that are significantly enriched as compared with the human genome. Bar height corresponds with percentage of all proteins found in that Protein Class. (See Additional file 2 for methods and detailed bioinformatics functional analysis).

In the next phase of our study we have combined the new methods for screening samples with well preserved synaptic proteomes with affinity methods to isolate MAGUK Associated Signalling Complexes (MASC). This affinity method relies on a 6 amino acid immobilized peptide (pep6, SIESDV) that binds to PDZ domains of MAGUK proteins and a control peptide (pep6δV, SSIESD) that cannot bind to these domains [24]. We used this protocol, which involves the use of stringent biochemical methods, to ask if it was possible to isolate MASC from 3 PM human frontal cortex samples with HUSPIR >1 (Additional file 1). As a positive control we used the same protocol on 3 NSB samples and as a further positive control, we also isolated MASCs from mouse cortex. As shown in the coomassie stained SDS-PAGE gel (Figure 3A), all samples showed strong recovery of multiple proteins with pep6, and as expected, a low background with pep6δV purifications. Immunoblotting of these samples with antibodies to three MAGUK proteins (DLG2/PSD93, DLG3/SAP102, DLG4/PSD-95) showed robust signals in pep6 samples and no signal in negative controls (Figure 3B). These results show that it is possible to affinity isolate MASCs from NSB as well as PM human brain and we hereafter refer to these isolates as hMASC.

We next identified all proteins in hMASC samples using mass spectrometry and used bioinformatics to compare PM and NSB components (Additional file 3). See Additional file 2 for detailed bioinformatics functional analysis of hMASC. In total we found 239 and 227 proteins in NSB and PM hMASC respectively. A very large proportion (>75%) of NSB hMASC proteins were found in PM hMASC complexes, demonstrating for the first time that it is possible to isolate *bona fide* synaptic multiprotein complexes from PM samples if these have a HUSPIR value >1. These results also indicate the applicability and robustness of the affinity isolation methods developed here to analyse human PM brain tissue. Comparison of the functional types of proteins in PM and NSB hMASC showed a high similarity (Figure 3C and Additional file 4).

Despite the high degree of similarity between NSB and PM hMASCs we reasoned that some protein types are likely more vulnerable to PM conditions than others. If this is the case it is important to identify them, as these will be underrepresented in PM hMASCs. We analysed several protein features, including protein length, isoelectric point, presence of transmembrane regions, and protein turn-over [25], in proteins isolated from both tissue sources. Overall, proteins found in NSB and PM hMASCs showed only minor differences among these variables. Only protein length was slightly biased, with proteins found in two or three triplicate samples somewhat longer in NSB hMASC compared to PM hMASC (median length 558, compared to 428 residues, respectively; p = 0.033, Mann Whitney Test). Next we compiled a list of the specific proteins with differential expression and noted this included proteins involved with activity-dependent reorganization of the synaptic proteome, including calcium-calmodulin dependent kinases (CAMK2A, B and G), scaffold proteins of the DLGAP family, G-proteins and elements of the tubulin cytoskeleton (Additional file 2: Figure S3). In contrast to these unstable components, the more stable groups included the MAGUK scaffold proteins, their associated NMDA receptors, signalling and cytoskeletal proteins, among others (Additional file 2: Figure S4).

### Human MASC proteins in disease

To understand the relevance of hMASC to human disease we searched online genetics databases and the published human genetics literature for evidence of hMASC genes involved in brain disorders (see methods). Our approach involved: i) identification of relevant genes and relevant diseases, ii) allocation of diseases into specific categories according to the International classification of Disease System (ICD-10), iii) statistical analysis of the representation of disease categories, and iv) comparison of hMASC with hPSD to examine the relative importance of hMASC, as compared with the whole postsynaptic density.

**Figure 4:**
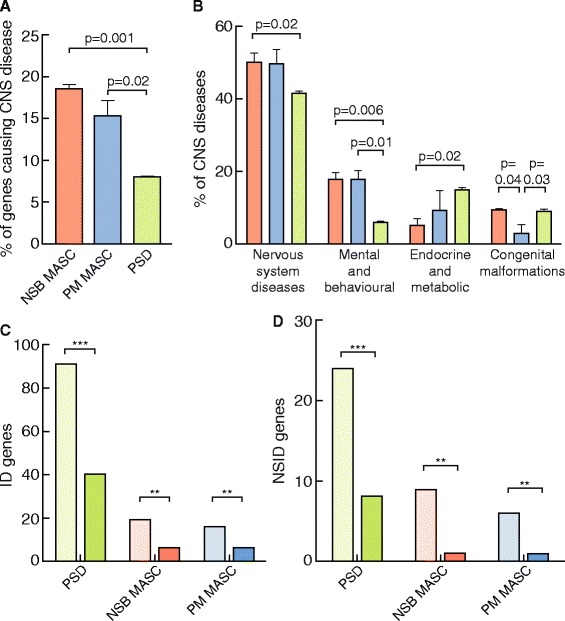
**Involvement of MASC proteins in genetic diseases. A**. Percentage of genes expressed in human NSB MASC, PM MASC and PSD causing central nervous system diseases included in OMIM. **B**. Proportion of CNS diseases caused by genes found in human NSB MASC (red), PM MASC (blue) and PSD (green). ICD-10 groups (Chapters) indicated on x-axis: Congenital Malformations refers to ICD-10 Chapter XVII, Nervous System Diseases refers to ICD-10 Chapter VI, Endocrine and metabolic refers to ICD-10 Chapter IV and Mental and Behavioural refer to ICD-10 Chapter V. Means compared by Student’s t-test. **C**. Genes expressed at the postsynapse causing any form of genetic Intellectual Disability (ID). For each postsynaptic protein complex pale columns indicate number of genes causing ID and dark columns the expected number to be found by chance. Binomial statistics are used to compute significance of the difference between observed and expected (Binomial Statistics, ***, p <1E-05; **, p <1E-03; *, p <0,05). **D**. Genes expressed at the postsynapse causing any form of genetic non-syndromic Intellectual Disability (NSID). For each postsynaptic protein complex pale columns indicate number of genes causing ID and dark columns the expected number to be found by chance. Binomial statistics are used to compute significance of the difference between observed and expected (Binomial Statistics, ***, p <1E-05; **, p <1E-03; *, p <0,05).

According to OMIM database of inherited diseases [26] 66 hMASC genes are involved with 93 genetic diseases. Fifty-one of these are nervous system diseases with 47 specific to the CNS. Literature text-mining and curation identified CNS diseases for which hMASC genes were positively associated, bringing the total number of CNS diseases related to the hMASC to 76. Classification with ICD-10 of these CNS diseases showed they include rare monogenic disorders as well as complex, more common psychiatric and neurologic disorders, including Schizophrenia and Autism spectrum disorder (Additional file 5). We next asked whether particular disease categories were over- or under-represented in hMASC compared to the postsynaptic proteome (hPSD, see Bayés et al. [2]). Looking into inherited diseases, we found a two-fold greater proportion of hMASC genes (both NSB and PM) involved with CNS compared with hPSD genes (Figure 4A). As shown in Figure 4B, the ‘Disorders of the Nervous System’ and ‘Mental and Behavioural Disorders’ ICD-10 categories were over-represented relative to the hPSD, whereas the hPSD was more enriched in Endocrine, Nutritional and Metabolic Diseases than hMASC (Figure 4B). As hMASC was most enriched for Mental & behavioural Disorders, we examined Intellectual Disability disorders in more detail. We created a database of genes causing any form of ID (ID) and non-syndromic ID (NSID) (Additional file 6) and found a significant over-representation of hMASC and hPSD genes in both these types of ID, with NSID highest (Figure 4C,D).

## Conclusions

We developed a simple method for determining if the synapse proteome integrity of a PM sample is sufficient for proteomics and rapid biochemical methods for the isolation of synapse-enriched fractions from small amounts of human PM tissue. In around two hours, synaptic proteomes can be obtained from as little as 100 mg of starting tissue, allowing for the parallel analysis of large numbers of samples. We have also shown that the HUman Synaptic Proteome Integrity Ratio (HUSPIR), an indicator of postsynaptic proteome integrity, denotes that the synaptic proteome is better conserved in cortical than other brain regions. Using these approaches we have also performed the first isolation and proteomic characterization of human MAGUK Associated Signalling Complexes and identified 76 CNS diseases involving MASC proteins, of which 47 are inherited disorders. The centrality of hMASC to human synapse function and human synaptic diseases is evidenced by the comparison of the hMASC and hPSD proteomes and their relevant genetic diseases.

Of the many diseases impacting on the synapse, hMASC is particularly important for those with cognitive components including ID, schizophrenia and autism. Recent whole exome analyses of schizophrenia show convergence of multiple mutations onto hMASC [15,16] and population studies show IQ is associated with genetic variation in hMASC genes [27]. Comparison of human and mouse synapse proteome orthologues shows a high degree of conservation in MASC and PSD proteins consistent with conserved functions [2]. Moreover, comparative cognitive testing with computerized touchscreen tests show MAGUK proteins in both species have conserved regulation of cognitive components [21].

Future studies on human synapse proteomes could use the Human Synapse Proteome Integrity Ratio (HUSPIR) to identify PM samples suitable for sophisticated proteomics from the thousands of stored brain bank samples. We were surprised to find that ~30% of cortical samples in the Edinburgh Brain Bank met the criteria of a HUSPIR >1, which suggests that large numbers of samples are suitable for analysis. Quantitative analysis of whole synapses or their subcomponents including hMASC can now be applied on a large scale to characterize human synaptopathies directly in human tissue. In addition to the use of HUSPIR, the identification of stable proteins indicates that these proteins and their relevant diseases will be better suited for study than those involving labile proteins. Synapse proteomics has identified a striking number of diseases that impact on the synapse and further development of methods for the study of human synaptopathy may have a major impact on neuropathology and diagnosis in the future. All the data generated in this study is freely available in the G2Cdb database (http://www.genes2cognition.org/publications/human-masc).

## Methods

### Human neocortex samples

NSB samples were obtained from frontal cortex by Prof. Ian Whittle (Edinburgh University) in a study approved by the Lothian Region Ethics Committee (/2004/4/16). Informed consent was obtained from all donors. All PM tissue was obtained from the Medical Research Council Edinburgh Brain Bank (Additional file 1). The three PM samples used to purify MASC were from the frontal cortex (Additional file 1).

### Rapid isolation of postsynaptic fractions from PM tissue

To facilitate characterization of the human postsynaptic proteome we developed biochemical methods based on those used in rodents [28,29]. Approximately 300 mg of grey matter was routinely used for isolation of synaptic fractions; nevertheless, the protocol can be scaled down to 100 mg of starting material. Prior to tissue processing, white matter was removed from frozen cortex blocks. Grey matter was homogenized with a glass-teflon dounce (tissue weight: volume ratio of 100 mg:1 ml) in a buffer containing 0.32 M sucrose, 10 mM HEPES pH 7.4, 2 mM EDTA, 5 mM sodium o-vanadate, 30 mM NaF and protease inhibitors cocktail (Roche). Homogenized tissue was centrifuged at 800 × g for 15 min at 4°C; supernatant removed and centrifuged at 10,000 × g for 15 min. The pellet was resuspended in Triton buffer (50 mM HEPES pH 7.4, 2 mM EDTA, 5 mM EGTA, 5 mM sodium-O-vanadate, 30 mM NaF, 1% Triton X-100 and protease inhibitors cocktail (Roche)) with half the volume used for homogenization. Sample was then centrifuged at 30,000 × g for 30 min at 4°C and the final pellet was resuspended in 200 μl of SDS buffer (50 mM Tris pH 7.4, 1% SDS) and used for immunoblotting.

### Isolation of postsynaptic densities from human post-mortem brain samples

Postsynaptic densities were isolated using standard differential ultracentrifugation methods. Briefly, dissected tissues was homogenized in buffer A (0.32 M sucrose, 2 mM HEPES, pH 7.4, EDTA-free protease inhibitor cocktail (Roche), phosphatase inhibitor cocktail set II (Calbiochem) using a Dounce homogenizer. The resulting homogenate was centrifuged at 1000 × g for 10 min at 4°C. The pellet was rehomogenized in the same buffer and centrifuged at 1000 × g for 10 min at 4°C. The two supernatants were pooled and centrifuged at 18600 × g for 15 min at 4°C. The next pellet was resuspended in buffer B (1.5 M sucrose and 50 mM Tris–HCl, pH 7.4 containing the same protease and phosphatase inhibitors as in buffer A). A discontinuous sucrose gradient was built by layering equal volumes of the resuspended pellet (bottom), 0.85 M and 0.3 M sucrose in 50 mM Tris–HCl, pH 7.4. This gradient was centrifuged at 60000 × g for 42 min using a MLA-55 rotor (Beckman-Coulter). The interface between 1.5 M and 0.85 M sucrose, corresponding to the synaptosomal fraction, was collected, diluted in 2 volumes of 50 mM Tris–HCl, pH 7.4 and centrifuged at 48000 × g for 30 min at 4°C. The resulting pellet was resuspended in 1.5% Triton X-100, 25 mM Tris–HCl, pH 7.4, incubated on ice for 30 min, layered on top of a 0.85 M sucrose solution in 50 mM Tris–HCl, pH 7.4 and centrifuged at 104000 × g for 30 min at 4°C. The resulting postsynaptic density pellet was resuspended in 50 mM Tris–HCl, pH 7.4 and 10% glycerol and mixed with SDS-PAGE electrophoresis loading buffer.

### Isolation of human MAGUK Associated Signalling Complexes (MASC)

MASCs were isolated from human NSB or PM frontal cortex using an improved version of an affinity purification protocol developed with mouse brain samples [12]. Briefly, this method used a peptide corresponding to the six carboxyl terminal residues of GluN2A and GluN2B, which bind PDZ domains of Membrane Associated Guanylate Kinase (MAGUK) proteins (DLG4/PSD95, DLG2/PSD93, DLG3/SAP102 & DLG1/SAP97) [30,31]. A negative control for the affinity purification uses a peptide lacking the C-terminal valine residue [24]. Brain tissue was homogenized (tissue weight:volume ratio of 100 mg:1.85 ml) with DOC Buffer (Tris 50 mM pH 9.0, 1% sodium deoxycholate, 50 mM NaF, 20 μM ZnCl_2_, 1 mM Sodium o-vanadate and protease inhibitors cocktail (Roche). The homogenized sample was centrifuged at 50,000 g for 30 min and pellet discarded. For affinity purification, peptides were first bound to Affi-Gel 10 resin (Bio-Rad) at a 5 mg/ml concentration following manufacturers indications. Brain homogenate and peptide-bound resin were mixed at 1:100 (v/v) ratio and incubated over-night at 4°C with agitation. Prior to elution, resin was washed five times with 5 column volumes of DOC Buffer. Elution was performed with DOC Buffer containing free peptide at 5 mg/ml. Eluted sample was reduced by adding fresh DTT (SIGMA) to a final concentration of 1 mM and heating at 70°C for 10 min. Reduced sample was carboxymethylated by adding fresh iodoacetamide (SIGMA) to final concentration of 2 mM and incubated in the dark at room temperature for 30 min.

### Sample preparation for quantitative MS

In gel digestion was performed as reported previously [2]. Extracted peptides were isotope labelled using a triplex dimethylation strategy [32,33] and labelled peptides pooled for LC-MS/MS analysis. This was done as follows: Light labelling: Neg. Control (50% from biopsy and 50% from PM Pep6δV), Medium labelling: Biopsy Pep6, Heavy labelling: PM Pep6. Pooled peptide samples from each of the 12 gel bands were analysed separately by nanoLC-MS/MS on a LTQ Orbitrap Velos (Thermo Fisher) hybrid mass spectrometer equipped with a nanospray source, coupled with an Ultimate 3000 Nano/Capillary LC System (Dionex). The system was controlled by Xcalibur 2.1 (Thermo Fisher) and DCMSLink 2.08 (Dionex). Peptides were desalted on-line using a micro-Precolumn cartridge (C18 Pepmap 100, LC Packings) and then separated using a 60 min RP gradient (4-32% acetonitrile/0.1% formic acid) on a BEH C18 analytical column (1.7 μm, 75 μm id × 10 cm,) (Waters). The LTQ-Orbitrap Velos was operated with a cycle of one MS (in the Orbitrap) acquired at a resolution of 60,000 at m/z 400, with the top 10 most abundant multiply-charged (2+ and higher) ions in a given chromatographic window subjected to MS/MS fragmentation in the linear ion trap. An FTMS target values of 1e^6^ and an ion trap MSn target value of 1e^4^ was used and with the lock mass (445.120025) enabled. Maximum FTMS scan accumulation time of 250 ms and maximum ion trap MSn scan accumulation time of 100 ms were used. Dynamic exclusion was enabled with a repeat duration of 45 s with an exclusion list of 500 and exclusion duration of 30 s.

MS data was analysed using MaxQuant [34] version 1.1.1.36. Data was searched against a Human IPI sequence database (v3.68) using following search parameters: trypsin with a maximum of 2 missed cleavages, 7 ppm for MS mass tolerance, 0.5 Da for MS/MS mass tolerance, with Acetyl (Protein N-term) and Oxidation (M) set as variable modifications and carbamidomethyl (C) as a fixed modification. A protein FDR of 0.01 and a peptide FDR of 0.01 were used for identification level cut offs. Protein quantification based on calculation of medium/light, heavy/light and heavy/medium dimethylated peptide ratios was performed using razor and unique peptides. The ratio of peptide abundances between Pep6 and PepδV affinity purifications was calculated independently for each protein and replicate. For a protein to be considered as a true positive in a given replicate its enrichment ratio had to be above threshold which was the sum of the median ratio and its absolute deviation (Median + MAD) [35]. Proteins with an abundance ratio above the threshold in 2 or 3 replicates were considered true components regardless of the number of peptides they have been identified with. For proteins found only in one replicate with a ratio above the threshold, to be considered a true positive, it had to be identified with at least three peptides.

### Analysis of MASC genes causing genetic nervous system diseases

Gene and phenotype entries were automatically extracted from OMIM [26] using the mim2gene file linking via their Entrez Gene identifiers. Diseases caused by MASC proteins were further classified, using information in the OMIM entry (Additional file 5), into central nervous system (CNS), peripheral nervous system (PNS), both CNS and PNS, or other. All diseases affecting the CNS, regardless of presence or absence of PNS involvement, were then grouped into chapters of the International Classification of Disease (ICD-10) developed by the World Health Organization (WHO). Those OMIM diseases that could not readily be found in ICD-10 were assigned to one of the ICD-10 chapters based on their symptoms, if possible, or classified as No-ICD 10 correspondence. Diseases in chapter VI (Diseases of the Nervous System) were divided into more specific blocks within that chapter.

To analyse the relevance of postsynaptic genes in syndromic or non-syndromic intellectual disability (ID or NSID) a list of genes known to cause these disorders was generated (Additional file 6). To create this database all genes linked to the terms “Mental Retardation” or “Intellectual Disability” were extracted from GeneCards [36] and added to a list of genes causing NSID curated from the scientific literature. Binomial statistics were used to calculate enrichment of postsynaptic components in ID or NSID.

### Text-mining and expert curation of MASC gene mutation associations to human nervous system disorders

PubMed abstracts up to August 2014 were searched for Human MASC gene mutations associated to disease, using a natural language processing approach to information extraction provided in the Linguamatics I2E platform [37]. Disease and disorder ontology terms from the Medical Dictionary for Regulatory Activities dictionary (MedDRA, www.meddra.org) were combined with a flexible pattern capturing genetic mutation terms, such as mutation, polymorphism, deletion, variation, and amino acid substitution patterns such as ‘K483E’, requiring mutation terms to be local to the gene, but allowing the disease term to appear anywhere in the sentence. The resulting sentences were examined by a biologist, checking each for correct gene identity (disambiguation), relevance to human genetics and limiting to disorders of nervous system origin, using a simple accept/reject criterion. In the cases where many sentence ‘hits’ were obtained to a particular gene and disease term combination, a maximum of 10 documents were examined. Redundant disease terms, such as symptomatic terms that describe a part of a larger clinical syndrome or disease were manually removed, thus we report a single representative PubMed identifier for each gene mutation-disease association combination. We then combined the curated text-mined PubMed search results with the OMIM extraction results, to produce an overview of the relationship between reported Human MASC gene mutations and nervous system disorders.

## Additional files

Additional file 1
**Human Samples.** Description of human samples used in this study. Additional file 2
**Additional information, figures and figure legends on immunoblot analysis of post-mortem brain and bioinformatics functional analysis of MASC complex.**
Additional file 3
**Proteomic Data.** List of proteins identified in the human MASC from neurosurgical and post-mortem tissue. Additional file 4
**Bioinformatics Functional Analysis.** Bioinformatics functional analysis of proteins identified in human MASC from neurosurgical and post-mortem tissue. Additional file 5
**MASC genes in Disease.** List of human MASC genes known to cause inherited diseases or positively associated with genetically complex psychiatric and neurologic disorders. Additional file 6
**MASC genes causing Intellectual Disability.** List of human MASC genes known to cause Intellectual Disability.

## Abbreviations

BA: Broadmann area
CNS: Central nervous system
hMASC: Human MASC
hPSD: Human PSD
HUSPIR: Human synapse proteome integrity ratio
ICD-10: International Classification of Disease, version 10
ID: Intellectual disability
MAGUK: Matrix associated guanilate Kinase
MASC: MAGUK associated signalling complex
NMDAR: N-methyl-D-aspartate receptor
NSB: Neurosurgical biopsy
NSID: Non-syndromic intellectual disability
PM: Post-mortem
PSD: Postsynaptic density
RIN: RNA integrity number
SEM: Standard error of the mean
