# Supplementary material for: Human post-mortem synapse proteome integrity screening for proteomic studies of postsynaptic complexes
Source: Mol Brain. 2014 Nov 28;7:88. doi: 10.1186/s13041-014-0088-4 (PMC4271336; doi:10.1186/s13041-014-0088-4)
Supplement: Additional file 2 — Additional information, figures and figure legends on immunoblot analysis of post-mortem brain and bioinformatics functional analysis of MASC complex. [file 13041_2014_88_MOESM2_ESM.docx]

### Additional file 1

### Methods

**Immunoblot and protein expression quantification.**

Protein concentration was determined using the Micro BCA protein assay kit (Pierce). For immunoblotting 10μg of total protein was resolved on 4–12% NuPage (Invitrogen) precast SDS-PAGE gels. Samples were transferred to PVDF membrane (Millipore) using a semi-dry system (Invitrogen) and probed with antibodies. The antibodies used were: Abcam (ACTB, ref. ab3280; RAB3A, ref. ab3335; SYNGAP1, ref. ab3344; SYP, ref. ab23754), BD Bioscience (BAIAP2, ref. 611394; SAP97/DLG1, ref. 610874; GluN2B, ref. 610416; MPDZ, ref. 611558; NOS1, ref. 610308), Cell Signalling (COX IV-1, ref. 4850; GSK3β, ref. 9315; MAPK1/3, ref. 4695), Merck Millipore (CAMK2A, ref. 05-532; GluN1, ref. 05-432; GRM1, ref.07-617; RAC1, ref. 05-389; YWHAZ, ref. 06-351), NeuroMab (PSD-93/DLG2, ref. 75-057; SAP102/DLG3, ref. 75-058), Santa Cruz (SHANK2, ref. sc-23545), Thermo Science (PSD-95/DLG4, ref. MA1-045) and US Biologicals (PRKCE, ref. P9103-20C). Following incubation with HRP-conjugated secondary antibodies the blots were developed using chemiluminescent reagent ECL Advanced (GE Healthcare).

Protein expression levels were quantified using ImageJ software. For analysis of postsynaptic proteins found in PM synaptic fractions, the intensity of the band of each component was measured and normalized to the band of the same component from NSB sample (NSB) that was run on each gel (Figure S1 and S2). From the screen of 20 postsynaptic proteins, the number detected in each sample was counted and correlated with brain quality markers using the Spearman correlation coefficient.

### MASC Bioinformatics Functional Analysis

Functional analyses were done using the Panther 9.0 Classification System [1], the Panther term ‘Protein Class’ and the Gene Ontology (GO) terms ‘Cellular Components’ and ‘Biological Process’. Binomial statistics were used to find enriched terms amongst components of postsynaptic protein complexes. To correct for multiple testing the Benjamini-Hochberg false discovery rate procedure was applied. Enrichment analysis were done either using the human genome as a background set or a ‘Postsynaptic Proteome’ set formed by proteins identified in human and mouse PSD [2] plus MASC proteins reported in this study.

### Analysis of MASC interaction network

Protein-protein interaction maps were obtained using STRING (v9.1) [3]. Only MASC proteins belonging to the NSB or PM High Confidence sets were considered in this analysis. Only high confidence interactions as defined by STRING (cut-off > 0,7) were used. Proteins were clustered using the MCL algorithm. Polyubiquitin C (UBC) was removed from list of NSB MASC – HC proteins prior to the analysis.

**Supplementary Results**

**Bioinformatic functional analysis of human MASC.**

In mice, MASC represents around 15% of the postsynaptic proteome of excitatory synapses and contains many proteins with fundamental roles in synaptic physiology and behaviour. This first proteomic data on human MASC (hMASC) allowed us to directly examine its composition and function with previously published proteomics of the human PSD proteome. hMASC proteins were classified into functional classes using the Panther Classification System [1] (Additional File 4) and enrichment of these classifications in MASC were identified and compared to the whole human genome (Figure 3c). As expected from previous studies in mice, hMASC complexes were enriched in glutamate receptors and scaffolding molecules (Figure 3c). Other PSD functions were depleted from hMASC including maintenance of the synaptic junction, endo/exocytosis and protein synthesis, all of which are important mechanisms for synaptic plasticity [4]. Furthermore, protein kinases and phosphatases were not enriched in hMASC in keeping with their low representation in mice (only 3 kinases and 3 phosphatases are found in the mouse complex of PSD-95 [5]), interestingly the types of kinases present in NSB and PM hMASCs differed (Figure S3). In contrast to the kinases, G-Proteins and Small GTPases were enriched in both NSB and PM hMASCs and the postsynaptic proteome as a whole. This data suggests that hMASC is more directly involved in processes that signal through these molecules.

**Supplementary Figures**


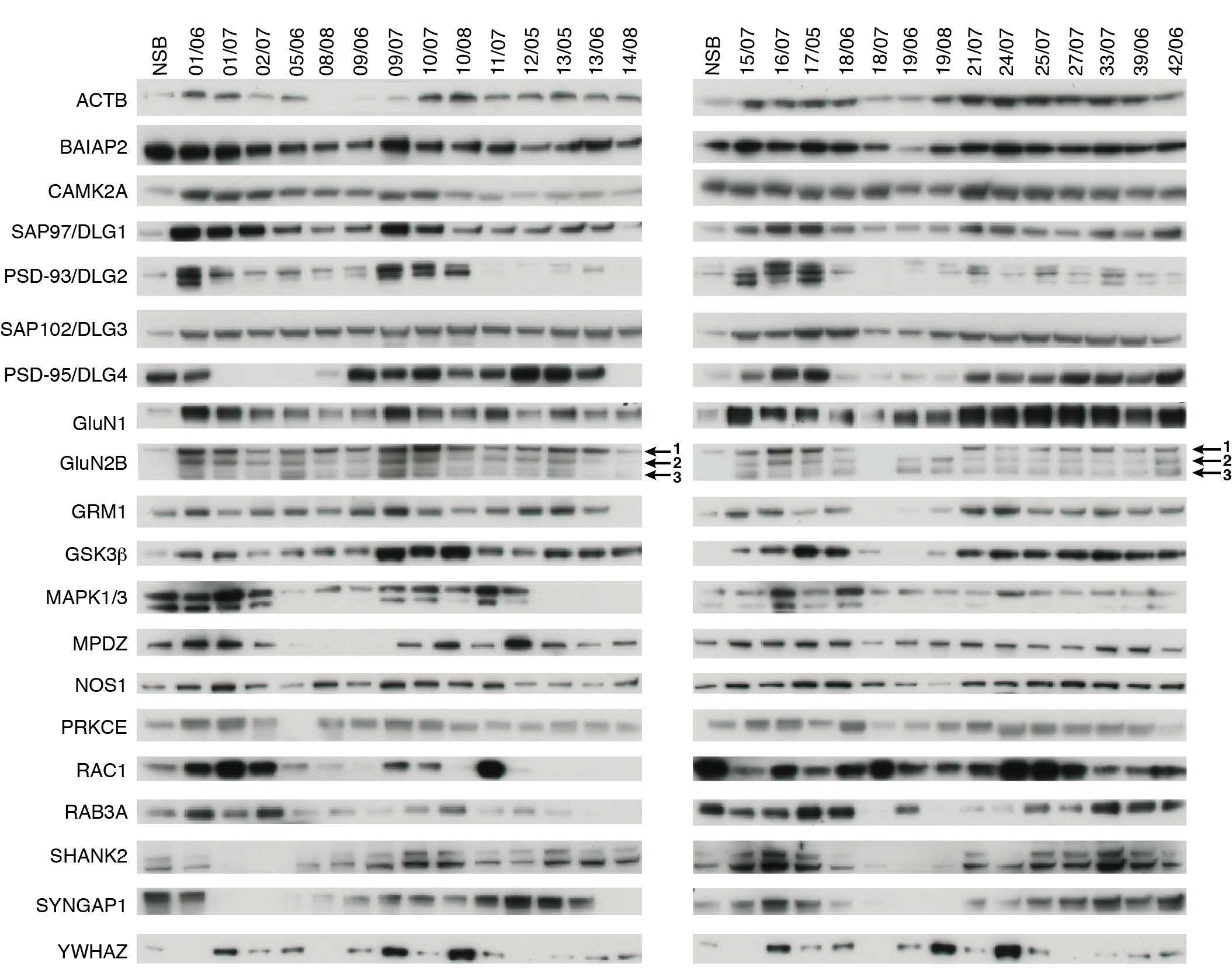


**Figure S1. Immunoblots of human PM samples with postsynaptic density proteins.**

Immunoblots of 20 different postsynaptic density components in synaptic-enriched fractions (P2) from 28 human PM frontal cortex samples. The reference codes for each sample are shown above each column. The first lane is a positive control of synaptic-enriched fraction from an NSB sample. Protein names are indicated at the left of each row. The three major GluN2B bands, including 2 degradation bands, are indicated by arrows.

**
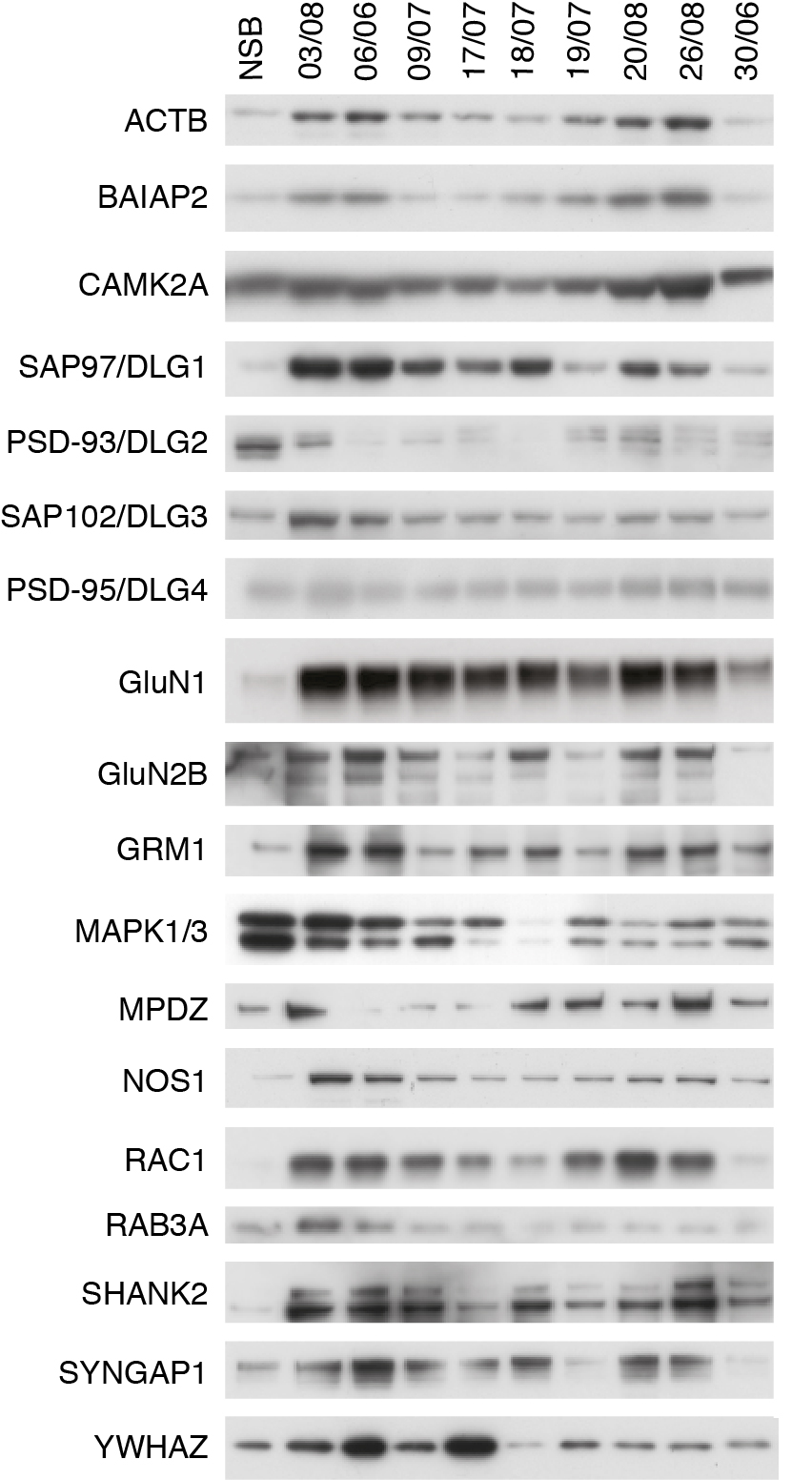
**

**Figure S2. Immunoblots of the selected group of human PM samples with postsynaptic density proteins.**

Immunoblots of different postsynaptic density components in synaptic-enriched fractions (P2) from 9 human PM cortex samples. The reference codes for each sample are shown above each column. The first lane is a positive control of synaptic-enriched fraction from an NSB sample. Protein names are indicated at the left of each row.

**
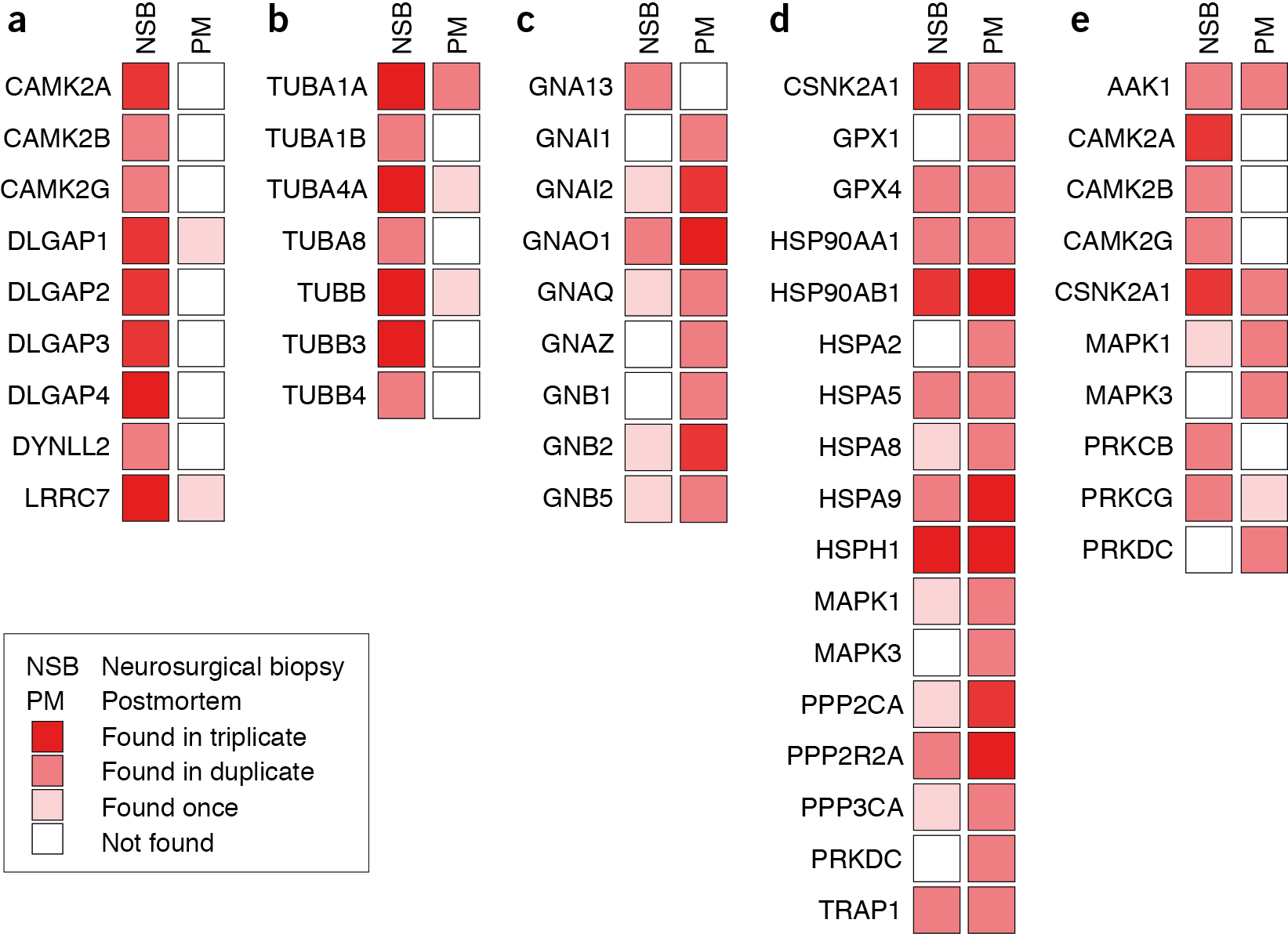
**

**Figure S3. Functional differences between hMASC isolated from NSB or PM tissue.**

Grid diagrams reflect whether a protein has been found in triplicate (bright red), duplicate (medium bright red), once (pale red) or have not been identified (white) in NSB or PM tissue.

**a.** Grid diagram for proteins from CAMK2 and DLGAP families, as well as DYNLL2 and LRCC7, known interactors of DLGAPs and CAMK2s, respectively.

**b.** Grid diagram showing proteins found in the Cellular component (GO) category ‘Tubulin Complex’, which is enriched in NSB hMASC as compared with the human genome.

**c.** Grid diagram showing proteins found in Panther Protein Class ‘Heterotrimeric G-proteins’, which is enriched in PM hMASC as compared with the human genome.

**d.** Grid diagram showing proteins found in the Biological Process (GO) category ‘Response to Stress’, which is enriched in PM hMASC as compared with the human genome.

**e.** Grid diagram showing proteins found under the term Protein Kinase in Panther Protein Class classification.


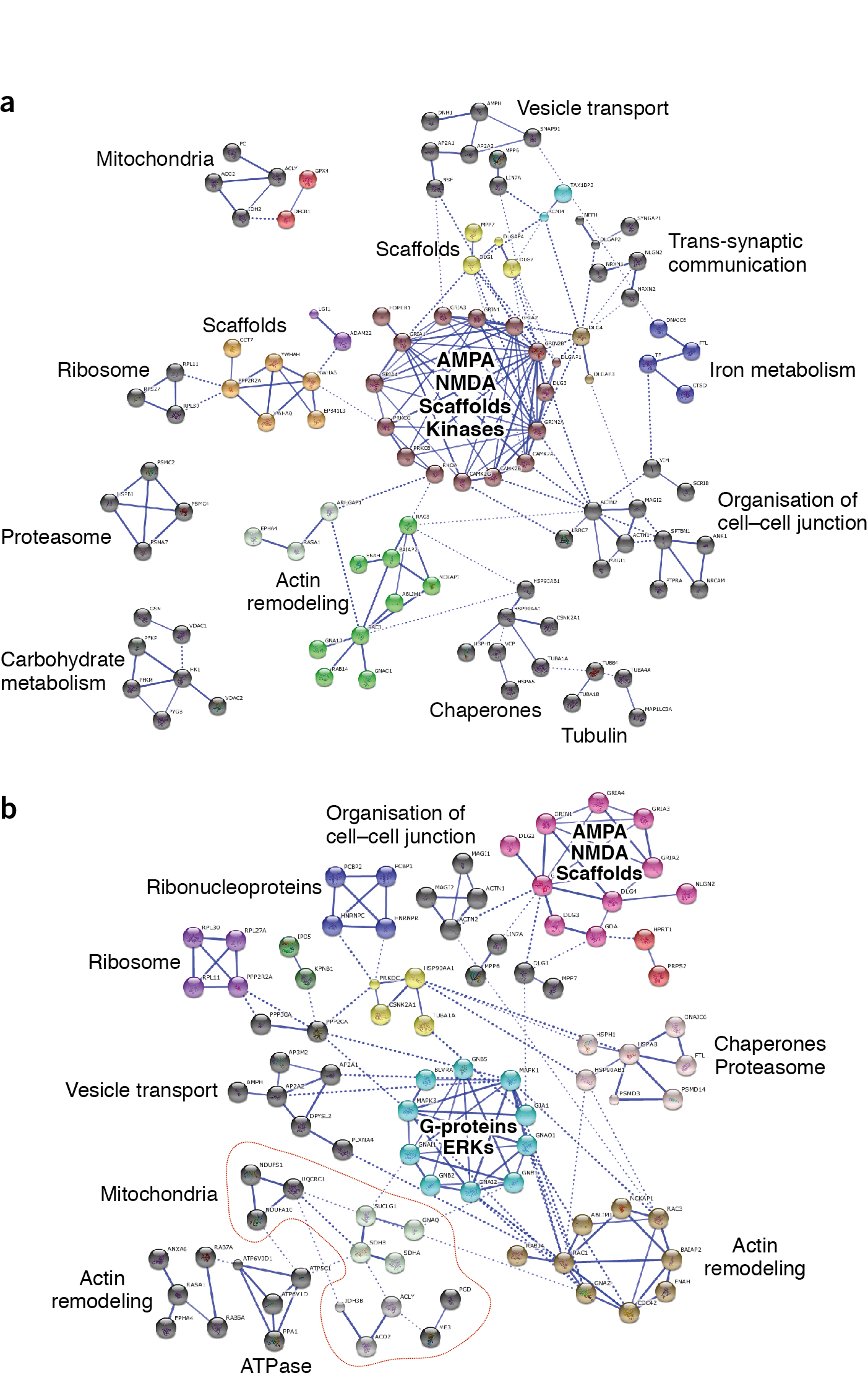


**Figure S4. MASC Interactome.**

**a.** Only protein identified with high confidence in NSB hMASC, that is, in triplicate or 2/3 times, were used to generate this interactome. Clusters of less than 4 proteins are not included in this image. Names next to the clusters are indicative of the Biological Function or Cellular Component common to most of its members.

**b.** Only protein identified with high confidence in PM hMASC, that is, in triplicate or 2/3 times, were used in this interactome. Clusters of less than 4 proteins are not included. Names next to the clusters are indicative of the Biological Function or Cellular Component common to most of its members.

**Supplementary References**

1. Mi H, Muruganujan A, Thomas PD: **PANTHER in 2013: modeling the evolution of gene function, and other gene attributes, in the context of phylogenetic trees.** *Nucleic Acids Research* 2013, **41**(Database issue):D377–86.

2. Bayés A, Collins MO, Croning MDR, Van De Lagemaat LN, Choudhary JS, Grant SGN: **Comparative study of human and mouse postsynaptic proteomes finds high compositional conservation and abundance differences for key synaptic proteins.** *PLoS ONE* 2012, **7**:e46683.

3. Franceschini A, Szklarczyk D, Frankild S, Kuhn M, Simonovic M, Roth A, Lin J, Minguez P, Bork P, Mering von C, Jensen LJ: **STRING v9.1: protein-protein interaction networks, with increased coverage and integration.** *Nucleic Acids Research* 2013, **41**(Database issue):D808–15.

4. Kandel ER: **The biology of memory: a forty-year perspective**. *J Neurosci* 2009, **29**:12748–12756.

5. Fernandez E, Collins MO, Uren RT, Kopanitsa MV, Komiyama NH, Croning MD, Zografos L, Armstrong JD, Choudhary JS, Grant SG: **Targeted tandem affinity purification of PSD-95 recovers core postsynaptic complexes and schizophrenia susceptibility proteins**. *Mol Syst Biol* 2009, **5**:269.
